# 6-Amino-1-benzyl-4-(4-chloro­phen­yl)-3-(4-pyrid­yl)-1,4-dihydro­pyrano[2,3-*c*]pyrazole-5-carbonitrile

**DOI:** 10.1107/S160053680800487X

**Published:** 2008-03-12

**Authors:** Frank Lehmann, Dieter Schollmeyer, Stefan Laufer

**Affiliations:** a Institute of Pharmacy, Department of Pharmaceutical and Medicinal Chemistry, Eberhard-Karls-University Tübingen, Auf der Morgenstelle 8, 72076 Tübingen, Germany; b Department of Organic Chemistry, Johannes Gutenberg-University Mainz, Duesbergweg 10-14, D-55099 Mainz, Germany

## Abstract

The crystal structure of the title compound, C_25_H_18_ClN_5_O, was determined in the course of our studies on the synthesis of 1,4-dihydro­pyrano[2,3-*c*]pyrazole as an inhibitor of the p38 mitogen-activated protein kinase (MAPK). The compound was prepared *via* a base-catalysed synthesis from 1-benzyl-3-(4-pyrid­yl)-1*H*-pyrazol-5(*4H*)-one with *p*-chloro­aldehyde and malononitrile. The crystal data obtained were used to generate a three-dimensional pharmacophore model for *in silico* database screening. The phenyl ring is disordered over two positions, with site occupancy factors of 0.55 and 0.45. The dihedral angles between the 1,4-dihydropyrano[2,3-*c*]pyrazole unit and the chloro­phenyl and pyridine rings are 83.7 (1) and 16.0 (1)°, respectively. The chloro­phenyl and pyridine rings make a dihedral angle of 86.8 (2)°.

## Related literature

The therapeutic potential of p38 mitogen-activated protein (MAP) kinase inhibitors for the treatment of inflammatory-associated diseases has been extensively reviewed (Kumar *et al.*, 2003 ▶; Pargellis & Regan, 2003 ▶). The synthesis of the title compound was performed according to the published procedures (Dyachenko & Chernega, 2005 ▶; Dyachenko & Rusanov, 2004 ▶; Klokol *et al.*, 1999 ▶).
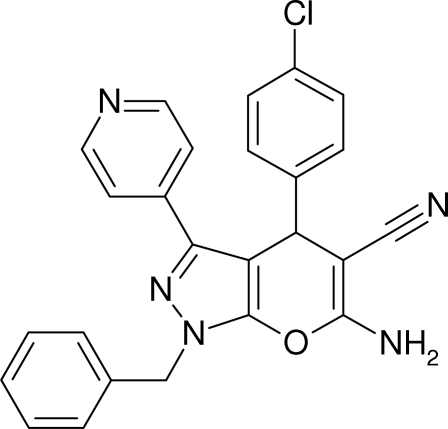


## Experimental

### 

#### Crystal data

C_25_H_18_ClN_5_O
*M* *_r_* = 439.89
Monoclinic, 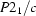

*a* = 5.7021 (11) Å
*b* = 17.795 (3) Å
*c* = 21.056 (9) Å
β = 90.954 (8)°
*V* = 2136.2 (11) Å^3^
*Z* = 4
Cu *K*α radiation
μ = 1.81 mm^−1^
*T* = 193 (2) K
0.46 × 0.12 × 0.08 mm

#### Data collection

Enraf–Nonius CAD-4 diffractometer
Absorption correction: ψ scan (*CORINC*; Dräger & Gattow, 1971 ▶) *T*_min_ = 0.825, *T*_max_ = 0.998 (expected range = 0.725–0.865)
4443 measured reflections
4018 independent reflections
2971 reflections with *I* > 2σ(*I*)
*R*_int_ = 0.026
3 standard reflections frequency: 60 min intensity decay: 2%

#### Refinement

*R*[*F* ^2^ > 2σ(*F* ^2^)] = 0.067
*wR*(*F* ^2^) = 0.203
*S* = 1.07
4018 reflections
307 parameters
66 restraints
H-atom parameters constrained
Δρ_max_ = 0.50 e Å^−3^
Δρ_min_ = −0.64 e Å^−3^

### 

Data collection: *CAD-4 Software* (Enraf–Nonius, 1989 ▶); cell refinement: *CAD-4 Software*; data reduction: *CORINC* (Dräger & Gattow, 1971 ▶); program(s) used to solve structure: *SIR92* (Altomare *et al.*, 1994 ▶); program(s) used to refine structure: *SHELXL97* (Sheldrick, 2008 ▶); molecular graphics: *PLATON* (Spek, 2003 ▶); software used to prepare material for publication: *PLATON*.

## Supplementary Material

Crystal structure: contains datablocks I, global. DOI: 10.1107/S160053680800487X/nc2090sup1.cif

Structure factors: contains datablocks I. DOI: 10.1107/S160053680800487X/nc2090Isup2.hkl

Additional supplementary materials:  crystallographic information; 3D view; checkCIF report

## Figures and Tables

**Table 1:** Hydrogen-bond geometry (Å, °)

*D*—H⋯*A*	*D*—H	H⋯*A*	*D*⋯*A*	*D*—H⋯*A*
N17—H17*A*⋯N30^i^	0.91	2.08	2.955 (3)	162
N17—H17*B*⋯N19^ii^	0.94	2.15	3.068 (3)	165

Symmetry codes: (i) 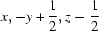; (ii) 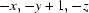.

**Fig. 1.**
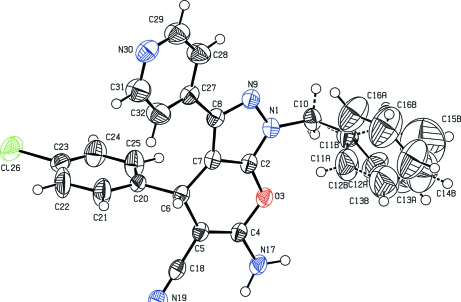
PLATON (Spek, 2003) view of 2. Displacement ellipsoids are drawn at the 50% probability level. H atoms are depicted as circles of arbitrary size.

**Fig. 2.**
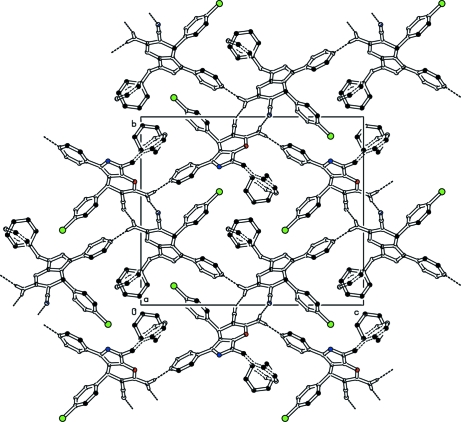
Crystal packing of compound 2. View along a axis. Only important H atoms are shown. Hydrogen bonds and disorederd phenyl rings are shown with dashed lines.

## supplementary crystallographic information

### Comment

p38 mitogen-activated protein (MAP) kinases are important enzymes in signal-transduction cascades which are responsive to stress stimuli, such as cytokines, ultraviolet irradiation, heat shock and osmotic shock, and are involved in cell transformation, proliferation, differentation and apoptosis. Until now there is a lack in new lead structures. Virtual screening of a database of compounds is one possibility to obtain new hints. The crystal structure of the title compound (Fig. 1) was used to consider a possible binding mode in the ATP pocket of the enzyme.

1,4-Dihydropyrano[2,3-*c*]pyrazole derivatives related to 2 have been published as crystal structures (Dyachenko & Rusanov, 2004). The nitrogen atoms N19 and N30 are involved in H-bond interactions with the amino group (N17) forming three-dimensional network (Fig. 2). The unsubstituted benzene ring is disordered in two orientations and was refined using a split model. The 1,4-Dihydropyrano[2,3-*c*]pyrazole moiety is almost planar and is nearly perpendiculare (83.7 (1)°) to the C20—C25 ring. The dihedral angle between the pyridine ring and the C20—C25 ring is 86.8 (2)°.

### Experimental

1-Benzyl-3-(4-pyridyl)-1*H*-pyrazol-5(4*H*)-one (1): A solution of ethyl 3-oxo-3-(4-pyridyl)propanoate (7.76 mmol) and triethylamine (7.76 mmol) in ethanol (15 ml) was cooled with an ice-bath. 2-Benzylhydrazinium chloride (7.76 mmol) was added and the reaction mixture was heated up to 60 °C for three hours. The solvent was evaporated to yield 98% of 1.

6-Amino-1-benzyl-4-(4-chlorophenyl)-3-(4-pyridyl)-1,4-dihydropyrano[2,3-*c*]pyrazole-5-carbonitrile (2): A mixture of *p*-chlorobenzaldehyde (0.80 mmol), malononitrile (0.80 mmol), *N*-methylmorpholine (0.80 mmol) in ethanol (10 ml) was stirred for one minute at room temperature. 1 was added and left to stand for one day. The precipitate formed was filtered and washed with ethanol and hexane. The compound was recrystallized from ethanol (31% yield).

### Refinement

Hydrogen atoms attached to carbons were placed at calculated positions with C—H=0.95A% (aromatic) or 0.99–1.00 Å (*sp*^3^ C-atom). Hydrogen atom attached to N17 were located in diff. fourier maps. All H atoms were refined with fixed isotropic thermal parameters using a riding motion model with *U*_iso_(H) = 1.2–1.5*U*_eq_ (parent atom). The phenyl ring C11 - C16 is disordered over two positions with s.o.f. of 0.55/0.45 and was refined as a rigid group.

### Crystal data

**Table:**

C_25_H_18_ClN_5_O	*F*_000_ = 912
*M**_r_* = 439.89	*D*_x_ = 1.368 Mg m^−^^3^
Monoclinic, *P*2_1_/*c*	Cu *K*α radiation λ = 1.54178 Å
Hall symbol: -P 2ybc	Cell parameters from 25 reflections
*a* = 5.7021 (11) Å	θ = 30–42º
*b* = 17.795 (3) Å	µ = 1.81 mm^−^^1^
*c* = 21.056 (9) Å	*T* = 193 (2) K
β = 90.954 (8)º	Needle, colourless
*V* = 2136.2 (11) Å^3^	0.46 × 0.12 × 0.08 mm
*Z* = 4	

### Data collection

**Table:**

Enraf–Nonius CAD-4 diffractometer	*R*_int_ = 0.026
Monochromator: graphite	θ_max_ = 69.9º
*T* = 193(2) K	θ_min_ = 3.3º
ω/2θ scans	*h* = 0→6
Absorption correction: ψ scan(CORINC; Dräger & Gattow, 1971)	*k* = 0→21
*T*_min_ = 0.825, *T*_max_ = 0.998	*l* = −25→25
4443 measured reflections	3 standard reflections
4018 independent reflections	every 60 min
2971 reflections with *I* > 2σ(*I*)	intensity decay: 2%

### Refinement

**Table:**

Refinement on *F*^2^	Secondary atom site location: difference Fourier map
Least-squares matrix: full	Hydrogen site location: inferred from neighbouring sites
*R*[*F*^2^ > 2σ(*F*^2^)] = 0.067	H-atom parameters constrained
*wR*(*F*^2^) = 0.203	*w* = 1/[σ^2^(*F*_o_^2^) + (0.0991*P*)^2^ + 1.7529*P*] where *P* = (*F*_o_^2^ + 2*F*_c_^2^)/3
*S* = 1.07	(Δ/σ)_max_ = 0.002
4018 reflections	Δρ_max_ = 0.50 e Å^−^^3^
307 parameters	Δρ_min_ = −0.64 e Å^−^^3^
66 restraints	Extinction correction: none
Primary atom site location: structure-invariant direct methods	

### Special details

**Table:**

Geometry. All e.s.d.'s (except the e.s.d. in the dihedral angle between two l.s. planes) are estimated using the full covariance matrix. The cell e.s.d.'s are taken into account individually in the estimation of e.s.d.'s in distances, angles and torsion angles; correlations between e.s.d.'s in cell parameters are only used when they are defined by crystal symmetry. An approximate (isotropic) treatment of cell e.s.d.'s is used for estimating e.s.d.'s involving l.s. planes.
Refinement. Refinement of *F*^2^ against ALL reflections. The weighted *R*-factor *wR* and goodness of fit *S* are based on *F*^2^, conventional *R*-factors *R* are based on *F*, with *F* set to zero for negative *F*^2^. The threshold expression of *F*^2^ > σ(*F*^2^) is used only for calculating *R*-factors(gt) *etc*. and is not relevant to the choice of reflections for refinement. *R*-factors based on *F*^2^ are statistically about twice as large as those based on *F*, and *R*- factors based on ALL data will be even larger.

### Fractional atomic coordinates and isotropic or equivalent isotropic displacement parameters (Å^2^)

**Table:**

	*x*	*y*	*z*	*U*_iso_*/*U*_eq_	Occ. (<1)
N1	0.9067 (5)	0.26498 (14)	0.08891 (12)	0.0361 (6)	
C2	0.7214 (5)	0.31100 (16)	0.08484 (13)	0.0336 (6)	
O3	0.6666 (4)	0.34475 (12)	0.02838 (9)	0.0367 (5)	
C4	0.4711 (5)	0.38996 (16)	0.03011 (13)	0.0328 (6)	
C5	0.3506 (5)	0.40201 (16)	0.08494 (13)	0.0332 (6)	
C6	0.4017 (5)	0.36554 (17)	0.14986 (13)	0.0327 (6)	
H6	0.2635	0.3341	0.1615	0.039*	
C7	0.6089 (5)	0.31463 (16)	0.14147 (13)	0.0330 (6)	
C8	0.7427 (5)	0.26449 (17)	0.18021 (14)	0.0340 (7)	
N9	0.9221 (5)	0.23499 (15)	0.14809 (12)	0.0372 (6)	
C10	1.0661 (6)	0.2425 (2)	0.03898 (16)	0.0438 (8)	
H10A	1.2188	0.2281	0.0585	0.053*	0.55
H10B	1.0933	0.2859	0.0106	0.053*	0.55
H10C	1.1120	0.2880	0.0152	0.053*	0.45
H10D	1.2100	0.2217	0.0593	0.053*	0.45
C11A	0.9722 (14)	0.1768 (3)	−0.0004 (3)	0.060 (5)	0.55
C12A	0.9179 (14)	0.1866 (3)	−0.0645 (3)	0.079 (2)	0.55
H12A	0.9415	0.2341	−0.0840	0.095*	0.55
C13A	0.8290 (16)	0.1269 (4)	−0.1000 (3)	0.106 (4)	0.55
H13A	0.7919	0.1336	−0.1438	0.127*	0.55
C14A	0.7945 (17)	0.0574 (4)	−0.0714 (4)	0.126 (5)	0.55
H14A	0.7338	0.0166	−0.0957	0.151*	0.55
C15A	0.8488 (17)	0.0477 (3)	−0.0074 (4)	0.134 (5)	0.55
H15A	0.8252	0.0002	0.0122	0.161*	0.55
C16A	0.9377 (16)	0.1074 (4)	0.0282 (2)	0.100 (3)	0.55
H16A	0.9748	0.1007	0.0719	0.120*	0.55
C11B	0.9766 (14)	0.1880 (5)	−0.0057 (4)	0.047 (4)	0.45
C12B	0.7484 (13)	0.1612 (6)	−0.0118 (4)	0.091 (4)	0.45
H12B	0.6351	0.1743	0.0189	0.110*	0.45
C13B	0.6861 (16)	0.1152 (6)	−0.0627 (5)	0.115 (5)	0.45
H13B	0.5302	0.0969	−0.0668	0.138*	0.45
C14B	0.852 (2)	0.0960 (7)	−0.1075 (5)	0.140 (7)	0.45
H14B	0.8094	0.0646	−0.1423	0.169*	0.45
C15B	1.080 (2)	0.1229 (8)	−0.1014 (6)	0.249 (14)	0.45
H15B	1.1936	0.1098	−0.1321	0.299*	0.45
C16B	1.1425 (14)	0.1689 (7)	−0.0505 (6)	0.122 (5)	0.45
H16B	1.2985	0.1872	−0.0464	0.146*	0.45
N17	0.4223 (3)	0.41620 (8)	−0.02841 (6)	0.0399 (6)	
H17A	0.5116	0.3977	−0.0603	0.060*	
H17B	0.2870	0.4458	−0.0355	0.060*	
C18	0.1547 (3)	0.45076 (8)	0.08023 (6)	0.0361 (7)	
N19	−0.0041 (3)	0.49044 (8)	0.07707 (6)	0.0465 (7)	
C20	0.4394 (3)	0.42549 (8)	0.20151 (6)	0.0333 (6)	
C21	0.2730 (3)	0.43846 (8)	0.24634 (6)	0.0660 (12)	
H21	0.1334	0.4093	0.2462	0.079*	
C22	0.3071 (9)	0.4943 (3)	0.2924 (2)	0.0825 (16)	
H22	0.1897	0.5039	0.3229	0.099*	
C23	0.5087 (8)	0.5345 (2)	0.29324 (17)	0.0548 (10)	
C24	0.6723 (8)	0.5225 (2)	0.2495 (2)	0.0604 (10)	
H24	0.8128	0.5512	0.2504	0.072*	
C25	0.6371 (7)	0.4684 (2)	0.20311 (18)	0.0543 (9)	
H25	0.7530	0.4610	0.1718	0.065*	
Cl26	0.5572 (3)	0.60204 (7)	0.35216 (6)	0.0929 (5)	
C27	0.7013 (6)	0.23447 (17)	0.24463 (14)	0.0368 (7)	
C28	0.8630 (7)	0.1865 (2)	0.27282 (18)	0.0548 (9)	
H28	1.0078	0.1767	0.2527	0.066*	
C29	0.8138 (7)	0.1527 (2)	0.33022 (18)	0.0588 (10)	
H29	0.9275	0.1196	0.3482	0.071*	
N30	0.6181 (6)	0.16380 (18)	0.36153 (13)	0.0514 (8)	
C31	0.4668 (7)	0.2105 (3)	0.33473 (18)	0.0625 (11)	
H31	0.3261	0.2205	0.3567	0.075*	
C32	0.4972 (7)	0.2462 (2)	0.27684 (18)	0.0581 (10)	
H32	0.3786	0.2781	0.2598	0.070*	

### Atomic displacement parameters (Å^2^)

**Table:**

	*U*^11^	*U*^22^	*U*^33^	*U*^12^	*U*^13^	*U*^23^
N1	0.0401 (14)	0.0352 (13)	0.0331 (13)	−0.0005 (11)	0.0039 (10)	−0.0038 (10)
C2	0.0419 (17)	0.0304 (14)	0.0286 (14)	−0.0032 (12)	0.0020 (12)	−0.0020 (11)
O3	0.0438 (12)	0.0397 (11)	0.0268 (10)	0.0027 (9)	0.0048 (8)	−0.0002 (8)
C4	0.0413 (16)	0.0289 (14)	0.0282 (14)	−0.0028 (12)	0.0011 (12)	−0.0017 (11)
C5	0.0387 (16)	0.0333 (15)	0.0277 (14)	−0.0012 (12)	0.0030 (12)	−0.0006 (11)
C6	0.0382 (16)	0.0336 (15)	0.0265 (14)	−0.0035 (12)	0.0037 (11)	0.0002 (11)
C7	0.0377 (16)	0.0317 (14)	0.0297 (14)	−0.0038 (12)	0.0032 (12)	−0.0022 (11)
C8	0.0381 (16)	0.0321 (15)	0.0319 (15)	−0.0040 (12)	0.0010 (12)	−0.0003 (12)
N9	0.0420 (14)	0.0356 (13)	0.0341 (13)	−0.0002 (11)	0.0008 (11)	−0.0014 (10)
C10	0.0424 (18)	0.0462 (19)	0.0433 (18)	−0.0002 (15)	0.0096 (14)	−0.0066 (15)
C11A	0.096 (11)	0.045 (5)	0.040 (6)	−0.010 (5)	0.008 (5)	−0.024 (4)
C12A	0.119 (6)	0.067 (5)	0.052 (4)	0.008 (5)	−0.023 (4)	0.002 (4)
C13A	0.142 (8)	0.101 (7)	0.074 (6)	0.009 (6)	−0.036 (6)	−0.025 (6)
C14A	0.167 (9)	0.110 (8)	0.100 (7)	−0.041 (7)	−0.009 (7)	−0.030 (6)
C15A	0.187 (9)	0.098 (7)	0.116 (8)	−0.044 (7)	−0.008 (7)	−0.020 (6)
C16A	0.149 (8)	0.074 (5)	0.077 (5)	−0.039 (6)	−0.010 (5)	−0.005 (4)
C11B	0.042 (7)	0.048 (5)	0.050 (7)	0.003 (5)	0.003 (5)	0.003 (5)
C12B	0.077 (6)	0.090 (7)	0.107 (7)	−0.008 (5)	−0.003 (6)	−0.054 (6)
C13B	0.104 (8)	0.113 (8)	0.129 (9)	0.000 (7)	−0.007 (7)	−0.036 (7)
C14B	0.155 (11)	0.131 (10)	0.135 (11)	−0.040 (8)	0.000 (8)	−0.049 (8)
C15B	0.248 (16)	0.252 (16)	0.248 (16)	−0.014 (10)	0.019 (10)	−0.033 (10)
C16B	0.129 (9)	0.128 (9)	0.110 (8)	−0.033 (7)	0.036 (7)	−0.057 (7)
N17	0.0501 (16)	0.0441 (15)	0.0255 (12)	0.0034 (12)	0.0042 (11)	0.0018 (11)
C18	0.0428 (17)	0.0410 (17)	0.0248 (14)	−0.0051 (14)	0.0044 (12)	−0.0004 (12)
N19	0.0488 (17)	0.0581 (18)	0.0329 (14)	0.0104 (15)	0.0056 (12)	0.0024 (12)
C20	0.0396 (16)	0.0359 (15)	0.0244 (13)	0.0051 (13)	0.0006 (11)	−0.0005 (11)
C21	0.052 (2)	0.091 (3)	0.056 (2)	−0.008 (2)	0.0152 (18)	−0.032 (2)
C22	0.075 (3)	0.112 (4)	0.061 (3)	0.011 (3)	0.018 (2)	−0.046 (3)
C23	0.072 (3)	0.047 (2)	0.0450 (19)	0.0216 (19)	−0.0219 (18)	−0.0117 (16)
C24	0.069 (3)	0.048 (2)	0.064 (2)	−0.0149 (19)	−0.002 (2)	−0.0152 (18)
C25	0.059 (2)	0.051 (2)	0.053 (2)	−0.0166 (18)	0.0170 (17)	−0.0129 (17)
Cl26	0.1409 (12)	0.0647 (7)	0.0714 (7)	0.0405 (7)	−0.0474 (8)	−0.0352 (6)
C27	0.0437 (17)	0.0337 (15)	0.0330 (15)	−0.0048 (13)	−0.0019 (13)	0.0017 (12)
C28	0.054 (2)	0.062 (2)	0.049 (2)	0.0111 (18)	0.0050 (17)	0.0155 (17)
C29	0.065 (2)	0.062 (2)	0.049 (2)	0.008 (2)	0.0004 (18)	0.0187 (18)
N30	0.0636 (19)	0.0524 (18)	0.0380 (15)	−0.0055 (15)	−0.0010 (14)	0.0104 (13)
C31	0.061 (2)	0.081 (3)	0.046 (2)	0.008 (2)	0.0127 (18)	0.020 (2)
C32	0.058 (2)	0.070 (3)	0.047 (2)	0.015 (2)	0.0106 (17)	0.0203 (18)

### Geometric parameters (Å, °)

**Table:**

N1—C2	1.338 (4)	C11B—C16B	1.3900
N1—N9	1.357 (3)	C12B—C13B	1.3900
N1—C10	1.457 (4)	C12B—H12B	0.9500
C2—O3	1.364 (3)	C13B—C14B	1.3900
C2—C7	1.365 (4)	C13B—H13B	0.9500
O3—C4	1.376 (4)	C14B—C15B	1.3900
C4—N17	1.342 (3)	C14B—H14B	0.9500
C4—C5	1.370 (4)	C15B—C16B	1.3900
C5—C18	1.416 (3)	C15B—H15B	0.9500
C5—C6	1.537 (4)	C16B—H16B	0.9500
C6—C7	1.502 (4)	N17—H17A	0.9113
C6—C20	1.536 (3)	N17—H17B	0.9442
C6—H6	1.0000	C18—N19	1.1496
C7—C8	1.422 (4)	C20—C25	1.362 (4)
C8—N9	1.342 (4)	C20—C21	1.3687
C8—C27	1.481 (4)	C21—C22	1.401 (5)
C10—C11B	1.438 (7)	C21—H21	0.9500
C10—C11A	1.524 (5)	C22—C23	1.354 (7)
C10—H10A	0.9900	C22—H22	0.9500
C10—H10B	0.9900	C23—C24	1.338 (6)
C10—H10C	0.9900	C23—Cl26	1.746 (4)
C10—H10D	0.9900	C24—C25	1.385 (5)
C11A—C12A	1.3900	C24—H24	0.9500
C11A—C16A	1.3900	C25—H25	0.9500
C12A—C13A	1.3900	C27—C32	1.373 (5)
C12A—H12A	0.9500	C27—C28	1.383 (5)
C13A—C14A	1.3900	C28—C29	1.382 (5)
C13A—H13A	0.9500	C28—H28	0.9500
C14A—C15A	1.3900	C29—N30	1.320 (5)
C14A—H14A	0.9500	C29—H29	0.9500
C15A—C16A	1.3900	N30—C31	1.318 (5)
C15A—H15A	0.9500	C31—C32	1.387 (5)
C16A—H16A	0.9500	C31—H31	0.9500
C11B—C12B	1.3900	C32—H32	0.9500
			
C2—N1—N9	109.8 (2)	C14A—C15A—H15A	120.0
C2—N1—C10	128.6 (3)	C15A—C16A—C11A	120.0
N9—N1—C10	121.5 (3)	C15A—C16A—H16A	120.0
N1—C2—O3	119.7 (2)	C11A—C16A—H16A	120.0
N1—C2—C7	110.8 (3)	C12B—C11B—C16B	120.0
O3—C2—C7	129.5 (3)	C12B—C11B—C10	127.8 (6)
C2—O3—C4	114.1 (2)	C16B—C11B—C10	111.8 (6)
N17—C4—C5	128.3 (3)	C13B—C12B—C11B	120.0
N17—C4—O3	109.6 (2)	C13B—C12B—H12B	120.0
C5—C4—O3	122.1 (3)	C11B—C12B—H12B	120.0
C4—C5—C18	116.3 (2)	C12B—C13B—C14B	120.0
C4—C5—C6	126.4 (3)	C12B—C13B—H13B	120.0
C18—C5—C6	117.3 (2)	C14B—C13B—H13B	120.0
C7—C6—C20	113.6 (2)	C15B—C14B—C13B	120.0
C7—C6—C5	106.7 (2)	C15B—C14B—H14B	120.0
C20—C6—C5	111.0 (2)	C13B—C14B—H14B	120.0
C7—C6—H6	108.4	C14B—C15B—C16B	120.0
C20—C6—H6	108.4	C14B—C15B—H15B	120.0
C5—C6—H6	108.4	C16B—C15B—H15B	120.0
C2—C7—C8	102.5 (3)	C15B—C16B—C11B	120.0
C2—C7—C6	120.9 (3)	C15B—C16B—H16B	120.0
C8—C7—C6	136.6 (3)	C11B—C16B—H16B	120.0
N9—C8—C7	111.2 (3)	C4—N17—H17A	116.2
N9—C8—C27	117.1 (3)	C4—N17—H17B	119.7
C7—C8—C27	131.3 (3)	H17A—N17—H17B	123.3
C8—N9—N1	105.7 (2)	N19—C18—C5	179.30 (13)
C11B—C10—N1	116.0 (4)	C25—C20—C21	118.13 (16)
C11B—C10—C11A	8.3 (5)	C25—C20—C6	120.9 (2)
N1—C10—C11A	112.7 (4)	C21—C20—C6	120.99 (12)
C11B—C10—H10A	113.4	C20—C21—C22	120.5 (2)
N1—C10—H10A	109.1	C20—C21—H21	119.7
C11A—C10—H10A	109.1	C22—C21—H21	119.7
C11B—C10—H10B	100.8	C23—C22—C21	119.4 (3)
N1—C10—H10B	109.1	C23—C22—H22	120.3
C11A—C10—H10B	109.1	C21—C22—H22	120.3
H10A—C10—H10B	107.8	C24—C23—C22	120.6 (3)
C11B—C10—H10C	108.3	C24—C23—Cl26	119.6 (3)
N1—C10—H10C	108.3	C22—C23—Cl26	119.8 (3)
C11A—C10—H10C	116.4	C23—C24—C25	120.1 (4)
H10A—C10—H10C	100.5	C23—C24—H24	119.9
H10B—C10—H10C	8.5	C25—C24—H24	119.9
C11B—C10—H10D	108.3	C20—C25—C24	121.2 (3)
N1—C10—H10D	108.3	C20—C25—H25	119.4
C11A—C10—H10D	103.3	C24—C25—H25	119.4
H10A—C10—H10D	7.3	C32—C27—C28	116.5 (3)
H10B—C10—H10D	114.5	C32—C27—C8	123.2 (3)
H10C—C10—H10D	107.4	C28—C27—C8	120.0 (3)
C12A—C11A—C16A	120.0	C29—C28—C27	120.0 (4)
C12A—C11A—C10	120.2 (4)	C29—C28—H28	120.0
C16A—C11A—C10	119.8 (4)	C27—C28—H28	120.0
C11A—C12A—C13A	120.0	N30—C29—C28	123.8 (4)
C11A—C12A—H12A	120.0	N30—C29—H29	118.1
C13A—C12A—H12A	120.0	C28—C29—H29	118.1
C12A—C13A—C14A	120.0	C31—N30—C29	115.7 (3)
C12A—C13A—H13A	120.0	N30—C31—C32	125.0 (4)
C14A—C13A—H13A	120.0	N30—C31—H31	117.5
C13A—C14A—C15A	120.0	C32—C31—H31	117.5
C13A—C14A—H14A	120.0	C27—C32—C31	118.9 (4)
C15A—C14A—H14A	120.0	C27—C32—H32	120.5
C16A—C15A—C14A	120.0	C31—C32—H32	120.5
C16A—C15A—H15A	120.0		
			
N9—N1—C2—O3	−177.1 (2)	C13A—C14A—C15A—C16A	0.0
C10—N1—C2—O3	−1.6 (5)	C14A—C15A—C16A—C11A	0.0
N9—N1—C2—C7	1.2 (3)	C12A—C11A—C16A—C15A	0.0
C10—N1—C2—C7	176.6 (3)	C10—C11A—C16A—C15A	179.0 (7)
N1—C2—O3—C4	179.3 (3)	N1—C10—C11B—C12B	8.6 (10)
C7—C2—O3—C4	1.4 (4)	C11A—C10—C11B—C12B	76 (4)
C2—O3—C4—N17	−175.7 (2)	N1—C10—C11B—C16B	−178.7 (6)
C2—O3—C4—C5	2.8 (4)	C11A—C10—C11B—C16B	−111 (4)
N17—C4—C5—C18	−2.8 (4)	C16B—C11B—C12B—C13B	0.0
O3—C4—C5—C18	179.0 (2)	C10—C11B—C12B—C13B	172.3 (10)
N17—C4—C5—C6	174.6 (3)	C11B—C12B—C13B—C14B	0.0
O3—C4—C5—C6	−3.5 (5)	C12B—C13B—C14B—C15B	0.0
C4—C5—C6—C7	0.2 (4)	C13B—C14B—C15B—C16B	0.0
C18—C5—C6—C7	177.6 (2)	C14B—C15B—C16B—C11B	0.0
C4—C5—C6—C20	124.6 (3)	C12B—C11B—C16B—C15B	0.0
C18—C5—C6—C20	−58.0 (3)	C10—C11B—C16B—C15B	−173.4 (9)
N1—C2—C7—C8	−1.2 (3)	C4—C5—C18—N19	−160 (100)
O3—C2—C7—C8	176.8 (3)	C6—C5—C18—N19	22 (11)
N1—C2—C7—C6	177.1 (3)	C7—C6—C20—C25	48.8 (3)
O3—C2—C7—C6	−4.8 (5)	C5—C6—C20—C25	−71.5 (3)
C20—C6—C7—C2	−119.2 (3)	C7—C6—C20—C21	−132.64 (18)
C5—C6—C7—C2	3.6 (4)	C5—C6—C20—C21	107.0 (2)
C20—C6—C7—C8	58.5 (4)	C25—C20—C21—C22	−0.1 (3)
C5—C6—C7—C8	−178.8 (3)	C6—C20—C21—C22	−178.7 (3)
C2—C7—C8—N9	0.9 (3)	C20—C21—C22—C23	−1.4 (6)
C6—C7—C8—N9	−177.0 (3)	C21—C22—C23—C24	1.7 (7)
C2—C7—C8—C27	−171.3 (3)	C21—C22—C23—Cl26	−177.8 (3)
C6—C7—C8—C27	10.8 (6)	C22—C23—C24—C25	−0.4 (7)
C7—C8—N9—N1	−0.2 (3)	Cl26—C23—C24—C25	179.1 (3)
C27—C8—N9—N1	173.2 (2)	C21—C20—C25—C24	1.4 (5)
C2—N1—N9—C8	−0.6 (3)	C6—C20—C25—C24	180.0 (3)
C10—N1—N9—C8	−176.4 (3)	C23—C24—C25—C20	−1.2 (6)
C2—N1—C10—C11B	−75.7 (6)	N9—C8—C27—C32	−164.6 (3)
N9—N1—C10—C11B	99.3 (6)	C7—C8—C27—C32	7.2 (5)
C2—N1—C10—C11A	−84.0 (5)	N9—C8—C27—C28	9.2 (5)
N9—N1—C10—C11A	91.0 (4)	C7—C8—C27—C28	−179.0 (3)
C11B—C10—C11A—C12A	0(3)	C32—C27—C28—C29	0.5 (6)
N1—C10—C11A—C12A	115.3 (5)	C8—C27—C28—C29	−173.7 (3)
C11B—C10—C11A—C16A	−179 (4)	C27—C28—C29—N30	−0.7 (7)
N1—C10—C11A—C16A	−63.8 (6)	C28—C29—N30—C31	−0.3 (6)
C16A—C11A—C12A—C13A	0.0	C29—N30—C31—C32	1.6 (7)
C10—C11A—C12A—C13A	−179.0 (7)	C28—C27—C32—C31	0.6 (6)
C11A—C12A—C13A—C14A	0.0	C8—C27—C32—C31	174.6 (4)
C12A—C13A—C14A—C15A	0.0	N30—C31—C32—C27	−1.7 (7)

### Hydrogen-bond geometry (Å, °)

**Table:**

*D*—H···*A*	*D*—H	H···*A*	*D*···*A*	*D*—H···*A*
N17—H17A···N30^i^	0.91	2.08	2.955 (3)	162
N17—H17B···N19^ii^	0.94	2.15	3.068 (3)	165

Symmetry codes: (i) *x*, −*y*+1/2, *z*−1/2; (ii) −*x*, −*y*+1, −*z*.
